# Cloxacillin‐ and Terbinafine‐Associated Mixed Drug‐Induced Liver Injury: Contrasting Clinical Outcomes and Causality Assessment Using the Updated RUCAM

**DOI:** 10.1155/crhe/1078239

**Published:** 2026-10-25

**Authors:** Ester Oliveras Casòliva, Antonio Jesús Araujo Montero, Alejandra de Carvalho, Santiago Montull Morer, Pinelopi Arvaniti

**Affiliations:** ^1^ Department of Internal Medicine Liver Unit General Hospital of Granollers Barcelona Spain

**Keywords:** cholestasis, cloxacillin, drug-induced liver injury, terbinafine, updated RUCAM, vanishing bile duct syndrome

## Abstract

**Background:**

Idiosyncratic drug‐induced liver injury (DILI) is an uncommon but potentially severe adverse drug reaction. Although cloxacillin and terbinafine are long‐established, widely prescribed agents, both have been associated with mixed/cholestatic DILI, occasionally resulting in prolonged cholestasis or vanishing bile duct syndrome (VBDS). We report two cases with similar clinical and biochemical presentations but markedly different histological findings and long‐term outcomes, illustrating that the initial biochemical phenotype may not reflect the extent of bile duct injury or predict subsequent clinical evolution.

**Case Report:**

An 83‐year‐old man presented with jaundice, dark urine, and severe pruritus after receiving cloxacillin for 2 months to treat acute paronychia. A 62‐year‐old man developed similar symptoms following a 2 month course of terbinafine for a fungal infection. Both patients exhibited a mixed biochemical pattern of liver injury. Comprehensive diagnostic evaluation, including detailed medical history, abdominal imaging, viral and autoimmune serology, excluded alternative causes of liver injury. Causality assessment using the updated RUCAM classified both cases as probable DILI. Liver biopsy demonstrated extensive ductopenia with loss of interlobular bile ducts in more than 50% of portal tracts, consistent with VBDS in the cloxacillin case, whereas the terbinafine case showed intracanalicular cholestasis with preserved bile ducts. HLA genotyping identified the HLA‐B∗57:01 and HLA‐A∗33:01 susceptibility alleles, respectively. Both patients received supportive treatment with ursodeoxycholic acid, cholestyramine, and rifampicin. The first patient achieved complete biochemical recovery after 8 months, whereas the second fulfilled criteria for chronic DILI with persistent liver test abnormalities beyond 1 year.

**Conclusions:**

These cases illustrate marked clinicopathological heterogeneity despite a similar initial presentation and mixed DILI biochemical pattern. The contrast between biopsy‐proven VBDS followed by complete biochemical recovery and preserved bile duct architecture followed by chronic DILI demonstrates that the extent of histological bile duct injury and long‐term outcome cannot necessarily be inferred from the initial biochemical phenotype.

## 1. Introduction

Idiosyncratic drug‐induced liver injury (DILI) is an unpredictable adverse hepatic reaction triggered by prescription medications, as well as herbal and dietary supplements (HDS), occurring independently of dose or treatment duration [1]. Although relatively uncommon, with an estimated annual incidence of 14–19 cases per 100,000 inhabitants in Europe, DILI represents a major healthcare challenge [2]. According to data from the Spanish DILI Registry and the DILI Network, approximately 60% of patients require hospitalization, 1.5%–4% ultimately undergo liver transplantation, and 2%‐3% die from liver‐related complications [3, 4].

The vast majority of idiosyncratic DILI cases are caused by commonly prescribed medications. Antibiotics account for nearly 40% of cases, with amoxicillin‐clavulanate being the most frequent culprit drug in several studies [3–5]. Although the absolute risk of DILI following antibiotic exposure remains very low, advanced age, metabolic syndrome, obesity, pre‐existing liver disease, and polypharmacy significantly increase susceptibility and severity [6]. Moreover, the biochemical pattern of injury varies according to the offending drug; for example, antituberculosis agents typically produce a hepatocellular phenotype, whereas β‐lactam antibiotics more commonly induce cholestatic or mixed patterns. Compared with hepatocellular DILI, cholestatic injury is associated with lower short‐term mortality, but with a more prolonged clinical course and a higher risk of chronic liver dysfunction [4, 7]. In rare cases, persistent cholangiocyte injury progresses to vanishing bile duct syndrome (VBDS), characterized histologically by the loss of interlobular bile ducts and associated with persistent cholestasis, secondary biliary cirrhosis, and occasionally the need for liver transplantation [8].

The diagnosis of DILI remains challenging because of its heterogeneous clinical presentation, variable latency, and the absence of specific diagnostic biomarkers. Consequently, DILI is primarily a diagnosis of exclusion, requiring careful evaluation of competing aetiologies and a detailed medication history. Structured causality assessment tools, including the updated Roussel Uclaf Causality Assessment Method (RUCAM), provide a standardized approach to causality assessment, but cannot definitively establish the diagnosis [9]. Liver biopsy may be useful in prolonged or atypical cases to exclude alternative diagnoses, characterize the pattern of injury, and identify bile duct loss or evolving VBDS, while HLA genotyping may contribute to distinguish DILI from autoimmune hepatitis in selected cases [6].

Besides the absence of reliable predictors of DILI occurrence, accurate prediction of disease severity remains an unmet clinical need. Hy’s law is widely accepted as a predictor of severe hepatocellular DILI, liver failure, and liver‐related mortality. However, because it relies on aminotransferase elevation and hyperbilirubinemia, its applicability to predominantly cholestatic or mixed DILI is less clear, potentially leading to underestimation of disease severity in these patients [10].

Considering that no reliable predictors of idiosyncratic DILI currently exist, prevention relies on appropriate prescribing, awareness of drug‐specific hepatotoxicity, individualization of risk, and early recognition of liver injury. The widespread and long‐standing use of a medication should never be interpreted as evidence of complete safety. In this context, we present two cases of severe mixed DILI associated with cloxacillin and terbinafine, that showed remarkably similar clinical and biochemical presentations but markedly different histological findings and subsequent outcomes. The cloxacillin‐associated case showed extensive ductopenia consistent with VBDS but ultimately achieved complete biochemical recovery, whereas the terbinafine‐associated case showed cholestasis with preserved bile ducts yet evolved to chronic DILI. This apparently discordant relationship between histological severity and clinical outcome highlights the heterogeneous and unpredictable course of idiosyncratic DILI and, together with the occurrence of severe liver injury after exposure to widely used drugs, reinforces the importance of individualized prescribing and early recognition, particularly in older, polymedicated patients.

## 2. Case Description

### 2.1. Case 1

An 83‐year‐old man was admitted to our hospital for painless jaundice, dark urine, and intense pruritus of 2 weeks duration. His previous medical history revealed arterial hypertension treated with angiotensin receptor antagonists, heart failure treated with torasemide, diabetes mellitus Type 2 treated with metformin, and osteopenia under vitamin D supplementation. Clinical evaluation revealed profound jaundice, an aortic murmur of 2/6, and peripheral pitting oedema. His laboratory work‐up revealed altered liver biochemistry with a mixed pattern (AST 220 U/L, ALT 431 U/L, GGT 703 U/L, ALP 434 U/L), direct hyperbilirubinemia (total bilirubin 8.2 mg/dL), and normal INR 1.13.

An abdominal ultrasound was performed, detecting normal liver parenchyma, permeable portal and hepatic veins, and excluding cholelithiasis. Also, a CT scan was performed, excluding the presence of liver infiltration, pancreatic lesions, or enlarged lymph nodes. A detailed medical history excluded consumption of alcohol or HDS, while a detailed investigation of previous and active medications revealed treatment with cloxacillin during 2 months for acute paronychia, suspended approximately 1 month before admission. After an extensive investigation for other causes of acute liver injury (viral hepatitis A, B, C, E, CMV, EBV, HSV, and leptospirosis) and a negative autoimmune liver serology for antinuclear (ANA), antismooth muscle cell (SMA), soluble liver antigen (SLA), and antimitochondrial (AMA) antibodies, a mixed DILI associated with recent cloxacillin use was considered as the most probable diagnosis. The R ratio, calculated from paired ALT and ALP values at admission, was 3, consistent with a mixed pattern of liver injury, and received an updated RUCAM score of 7, indicating probable DILI.

Accordingly, symptomatic treatment with cholestyramine was initiated for the control of intense pruritus. However, during hospitalization the patient presented a progressive worsening of liver biochemistry (Figure 1A) reaching maximum bilirubin values of 17 mg/dL, prolongation of INR 1.3, and acute kidney injury KDIGO II, without signs of ascites or hepatic encephalopathy. Therefore, a liver biopsy was performed at 7 days of hospitalization (3 weeks from admission), revealing ductopenia with atrophic, or absent bile ducts in > 50% of portal tracts and marked canalicular cholestasis, consistent with VBDS (Figure 1B). HLA genotyping revealed the presence of HLA‐B∗57:01 allele, related with flucloxacillin toxicity and VBDS. UDCA was subsequently initiated with slow but progressive improvement of liver markers and normalization of liver biochemistry after 8 months of follow‐up.

**FIGURE 1:**
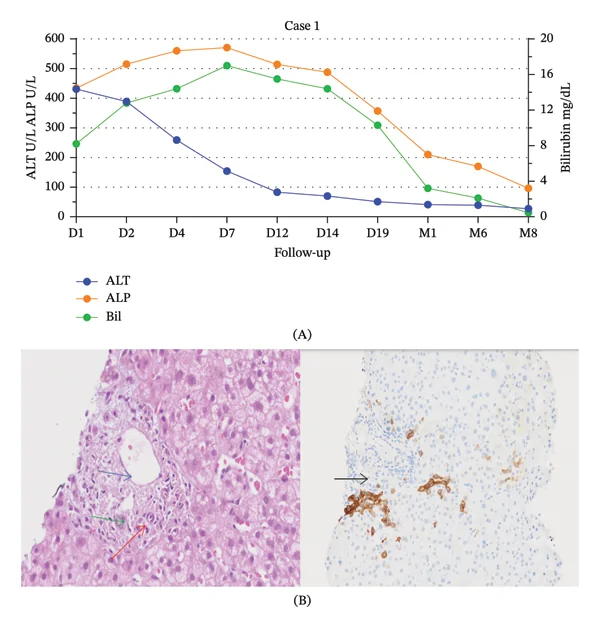
Biochemical evolution and liver histology of cloxacillin‐induced mixed DILI. (A) Serial evolution of serum ALT, ALP, and total bilirubin from hospital admission (D1) to complete biochemical recovery at month 8 (M8). (B) Liver biopsy containing 13 complete portal tracts, showing a portal tract with the hepatic artery (red arrow) and portal vein (blue arrow) with degenerative changes of bile ducts (green arrow). CK7 immunostaining demonstrates ductopenia (black arrow) with biliary metaplasia of periportal hepatocytes, consistent with vanishing bile duct syndrome. Abbreviations: D day, M month, Y year, ALT alanine aminotransferase, ALP alkaline phosphatase, Bil bilirubin, CK cytokeratin 7, DILI drug‐induced liver injury. Upper normal limits: ALT 40 U/L, ALP 120 U/L, bilirubin 1.2 mg/dL.

### 2.2. Case 2

The second case was a 62‐year‐old man, admitted in our department for painless jaundice and nonketotic hyperglycemia. His medical history consisted of diabetes mellitus Type 2 treated with metformin, SGLT2 inhibitors, and high doses of insulin, and obesity (BMI 32.6). Clinical evaluation revealed jaundice and central obesity. His laboratory work‐up revealed altered liver biochemistry with a mixed pattern (AST 572 U/L, ALT 677 U/L, GGT 1301 U/L, ALP 689 U/L), hyperbilirubinemia (total bilirubin 8.33 mg/dL) and normal INR 0.89.

Consumption of alcohol and HDS was excluded, while medical history revealed previous treatment with terbinafine during 2 months for cutaneous fungal infection, suspended 2 weeks before admission. An extensive diagnostic evaluation, including abdominal ultrasound showing moderate steatosis, viral and autoimmune serologies, and serum ceruloplasmin, excluded alternative causes. The R ratio at presentation was 2.9, indicating a mixed pattern of liver injury, most probably induced by terbinafine, and was classified as probable DILI according to the updated RUCAM score of 6.

During hospitalization the patient presented intense pruritus partially controlled with cholestyramine and worsening of liver biochemistry with maximum bilirubin levels at 18.5 mg/dL at 8 days of hospitalization (Figure 2A). Therefore, to complete the work‐up a liver biopsy was performed after 9 days of hospitalization revealing intracanalicular cholestasis with intact bile ducts and steatosis of less than 5%, without signs of steatohepatitis (Figure 2B). Interestingly, HLA genotyping revealed the presence of HLA‐A∗33:01 allele associated with terbinafine induced liver injury. Rifampicin was added for the control of intense pruritus and a progressive improvement of liver biochemistry was observed during the next 3 months from initial presentation, however without complete normalization of liver enzymes at 12 months of follow‐up.

**FIGURE 2:**
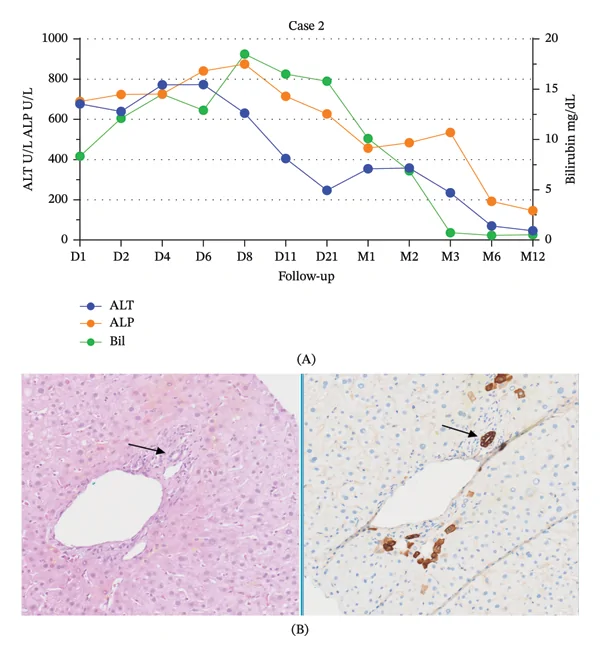
Biochemical evolution and liver histology of terbinafine‐induced mixed DILI. (A) Serial evolution of serum ALT, ALP and total bilirubin from hospital admission (D1) to 12 months (M12). Liver biochemistry progressively improved; however, persistent abnormalities beyond 12 months after DILI recognition fulfilled the criteria for chronic DILI. (B) Liver biopsy containing 11 complete portal tracts, demonstrating preserved interlobular bile ducts (black arrows) with intracanalicular cholestasis, without evidence of ductular reaction or ductopenia. Abbreviations: D day, M month, Y year, ALT alanine aminotransferase, ALP alkaline phosphatase, Bil bilirubin, CK cytokeratin 7, DILI drug‐induced liver injury. Upper normal limits: ALT 40 U/L, ALP 120 U/L, bilirubin 1.2 mg/dL.

## 3. Discussion

These two cases illustrate the heterogeneous clinical spectrum of mixed/cholestatic DILI caused by long‐established medications. Despite similar biochemical phenotypes and a probable causal relationship according to the updated RUCAM score, their histological findings and long‐term outcomes differed substantially, ranging from biopsy‐proven VBDS with complete biochemical recovery to chronic DILI with persistent liver test abnormalities beyond 1 year. Beyond their individual clinical interest, these cases convey two important messages for everyday clinical practice. First, the widespread use and familiarity of a medication should never be interpreted as evidence of complete safety. Second, the clinicopathological dissociation observed between the two cases illustrates the heterogeneity of DILI and suggests that neither the initial biochemical phenotype nor the presence of ductopenia in isolation necessarily determines subsequent clinical evolution.

Although idiosyncratic DILI is considered uncommon, its clinical impact is considerable. A population‐based study from Iceland demonstrated a marked age‐dependent increase in incidence, rising from fewer than 10 cases per 100,000 inhabitants among young adults to approximately 40 per 100,000 in individuals older than 70 years [11]. Likewise, prospective data from the Spanish DILI Registry showed that approximately 60% of patients require hospitalization, increasing to almost 73% among elderly patients presenting with jaundice and cholestatic or mixed liver injury [4]. These observations highlight that, despite its relatively low incidence, DILI represents a substantial healthcare burden, particularly in aging populations.

In keeping with these observations, both of our patients belonged to groups at increased risk of mixed/cholestatic DILI. The first patient was 83‐year‐old with multiple comorbidities and polypharmacy, whereas the second had obesity and Type 2 diabetes, representing a relevant metabolic risk profile. Data from the Spanish DILI Registry consistently demonstrate that increasing age is associated with a shift towards cholestatic and mixed injury patterns, and together with a higher prevalence of diabetes, hypertension, dyslipidaemia, and polypharmacy, it may influence susceptibility, phenotype, or outcome in selected drug‐specific settings [4, 6]. Although hepatocellular DILI remains the phenotype most strongly associated with liver‐related mortality, mixed/cholestatic injury is characterized by a more prolonged clinical course, increased risk of chronic liver dysfunction, and greater healthcare utilization [3, 7]. These findings support the need for individualized risk assessment before prescribing potentially hepatotoxic medications, particularly in elderly patients and those with significant metabolic comorbidities.

Regarding the culprit agents, cloxacillin and terbinafine are well‐recognized causes of idiosyncratic DILI, although they differ in their characteristic histopathological evolution. Although data are more robust in the case of flucloxacillin, cloxacillin has also been associated with prolonged cholestasis, ductopenia, and VBDS [12, 13], while terbinafine more commonly induces prolonged cholestatic hepatitis with or without bile duct injury [14]. Our findings closely mirror these established phenotypes, with biopsy demonstrating extensive ductopenia compatible with VBDS in the cloxacillin‐treated patient and intracanalicular cholestasis with preserved bile ducts following terbinafine exposure. Although neither liver biopsy nor HLA genotyping is routinely recommended for the diagnosis of DILI, both provided valuable complementary information in our patients. Histology not only excluded competing diagnoses, but also characterized the pattern and severity of bile duct injury, allowing distinction between established VBDS and preserved duct architecture. Furthermore, the identification of HLA‐B∗57:01 and HLA‐A∗33:01, respectively, supports their classification within the recently proposed HLA‐based immune idiosyncratic DILI subtype [15]. HLA‐B57:01 is a well‐established susceptibility allele for flucloxacillin‐induced DILI [16, 17]. Although this pharmacogenetic association should not be directly extrapolated to cloxacillin, mechanistic studies demonstrated that HLA‐B57:01‐restricted flucloxacillin‐responsive T‐cell clones cross‐react with cloxacillin and other isoxazolyl penicillins, providing biological plausibility for a shared immune mechanism [15]. Accordingly, HLA‐A33:01 01 has been strongly associated with terbinafine‐induced DILI, and this association was subsequently confirmed in a dedicated terbinafine cohort, in which 10/11 European‐ancestry patients carried HLA‐A∗33:01 [18, 19]. Thus, both cases were classified biochemically as mixed DILI and are consistent with the HLA‐based immune idiosyncratic DILI spectrum proposed in the refined immunological classification. Nevertheless, HLA genotyping remains supportive rather than diagnostic, and causality in both cases was established through the clinical evaluation and updated RUCAM.

In both patients, the updated RUCAM score supported a probable causal relationship and liver biopsy proved decisive in defining the extent of bile duct injury [9]. Conversely, Hy’s law could not be applied because neither patient fulfilled the criteria for hepatocellular injury, highlighting an important limitation of current prognostic tools, which were primarily developed for hepatocellular DILI and may underestimate the severity of cholestatic and mixed phenotypes [10]. Chronic DILI is defined as persistent abnormalities in liver biochemistry and radiological or histological evidence of ongoing liver injury 12 months after DILI recognition, according to the criteria proposed by the Spanish DILI Registry and endorsed by the EASL Clinical Practice Guidelines [6, 20, 21]. While the patient with biopsy‐proven VBDS eventually achieved complete biochemical recovery, his clinical course was complicated by severe hyperbilirubinemia, coagulopathy, and acute kidney injury, placing him at high risk of liver‐related complications [22]. In contrast, the second patient fulfilled the criteria for chronic DILI, with persistent liver test abnormalities beyond 12 months despite withdrawal of terbinafine. Whether metabolic comorbidities influenced the prolonged course of DILI remains uncertain; however, the presence of < 5% of steatosis in liver biopsy and the previously normal liver biochemistry suggest that DILI was the principal driver of persistent liver dysfunction.

In conclusion, these cases reinforce the need to maintain awareness of the hepatotoxic potential of widely used and long‐established medications, particularly in older, polymedicated patients in whom individual susceptibility and concomitant treatments may influence the clinical course. They also illustrate the limited ability of the initial clinical and biochemical presentation to anticipate subsequent histological findings and long‐term evolution, as more severe bile duct injury did not translate into a worse outcome in our patients. Careful prescribing, individualized risk assessment, and early recognition of liver injury therefore remain essential to minimize the risk of severe or persistent DILI.

## Funding

No funding was received.

## Consent

Written informed consent for publication of these cases was obtained from both patients. The manuscript has been prepared in accordance with ethical standards and contains no identifiable personal information or images. Patient anonymity has been preserved throughout the manuscript, with only nonidentifiable demographic information (age and sex) reported.

## Conflicts of Interest

The authors declare no conflicts of interest.

## Data Availability

The data supporting the findings of this case report are available from the corresponding author upon reasonable request. The data are not publicly available due to privacy or ethical restrictions.
